# Fractured Ribs and the CT Funky Fat Sign of Diaphragmatic Rupture

**DOI:** 10.1155/2016/6723632

**Published:** 2016-06-26

**Authors:** Iclal Ocak, Diane C. Strollo

**Affiliations:** ^1^University of Pittsburgh Medical Center, Radiology Suite 200 East Wing, 200 Lothrop Street, Pittsburgh, PA 15213, USA; ^2^University of Pittsburgh Medical Center, 200 Lothrop Street, Pittsburgh, PA 15213, USA

## Abstract

Traumatic diaphragmatic rupture remains a diagnostic challenge for both radiologists and surgeons. In recent years, multidetector CT has markedly improved the diagnosis of diaphragmatic injury in polytrauma patients. Herein, we describe two cases of subacute presentation of traumatic diaphragmatic rupture from a penetrating rib fracture and subsequent intrathoracic herniation of omental fat, representing the CT “funky fat” sign.

## 1. Introduction

Traumatic diaphragmatic rupture (TDR) may result from a penetrating injury or blunt thoracoabdominal trauma and results in communication between the pleural and peritoneal cavities [1–3]. A penetrating injury from a displaced rib fracture or a stab or gunshot wound typically causes a small diaphragmatic puncture [1–3]. With blunt trauma, a sudden increase of intra-abdominal pressure may cause a ≥10 cm “blowout” laceration of the diaphragm [4]. In some cases, both mechanisms may occur.

The fractured rib sign was first described by Holland and Quint [5] and later referred to as “presumed laceration of the diaphragm by a fractured rib” in a study by Nchimi et al. [6]. The fractured rib sign is present when a rib fragment points toward and directly penetrates the diaphragm. The reported sensitivity of the fractured rib sign is low, and specificity statistics have not been reported [6]. In patients with severe trauma, this sign should direct attention to the diaphragm and prompt close clinical and imaging follow-up [1].

Numerous signs indicating TDR have been described in the literature [1]. We present two patients with TDR with fractured rib sign on initial trauma multidetector computed tomography (MDCT) and subsequent herniation of omental fat, the funky fat sign, within two weeks of the injury.

## 2. Case  1

**Figure 1:**
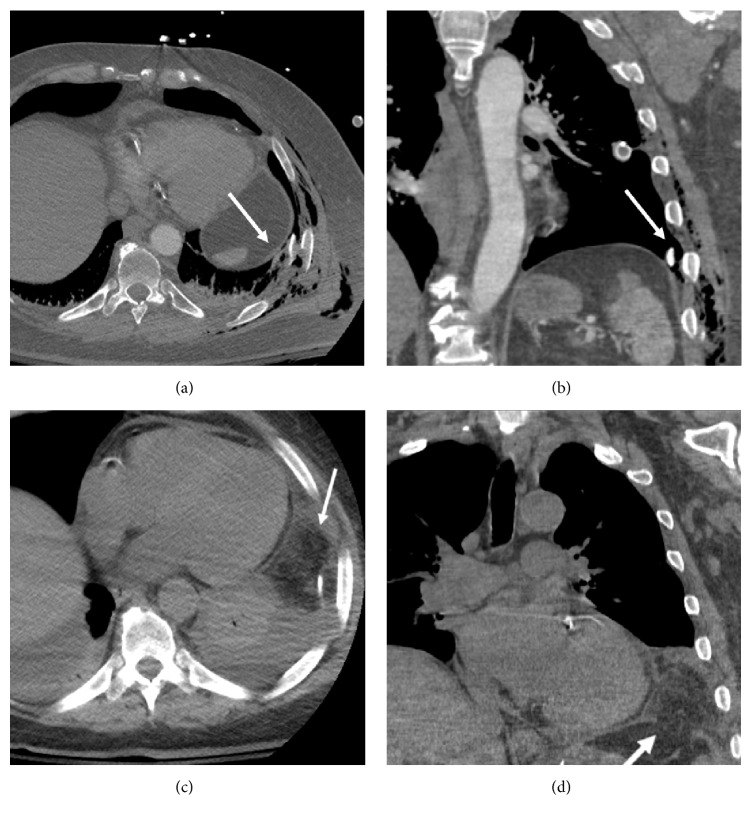
Fractured rib sign in a 59-year-old man after blunt trauma. (a, b) Contrast-enhanced chest MDCT (axial and coronal planes) shows left 10th-rib fracture abutting the diaphragm (fractured rib sign). The diaphragm appears intact on initial trauma CT. (c, d) Repeat chest MDCT (axial and coronal planes) 5 days later reveals new herniation of omental fat (arrows) into the left hemithorax due to subacute diaphragm rupture. Note discontinuity of the diaphragm (d).

A 59-year-old helmeted man was thrown 20 feet during a motorcycle collision. Because of multiple comminuted left rib fractures with subcutaneous gas on chest radiograph, a left chest tube was placed. Contrast-enhanced trauma MDCT revealed sternal and scapular fractures, left lung contusion, and a small left pneumothorax. In addition, multiple fractures of the 3rd through 10th left ribs were displacement into the chest cavity, with associated chest wall instability. While the 10th-rib fracture abutted the left diaphragm, the diaphragm appeared intact (Figures 1(a) and 1(b)). The patient did not require intubation. Repeat chest MDCT ten days later revealed herniation of omental fat into the left chest, consistent with subacute TDR (Figures 1(c) and 1(d)). However, this finding was missed until a third MDCT was performed three days later. Thoracoscopic exploration confirmed the TDR contained only omentum, which was edematous and viable but could not be completely reduced from the thoracic approach. Therefore, laparoscopic reduction of omental fat and repair of the diaphragm laceration were performed on hospital day 13. Because the left rib fractures had become progressively displaced, side plate fixation of the left 6–8th ribs was performed on hospital day 14. The patient had otherwise uneventful recovery.

## 3. Case  2

**Figure 2:**
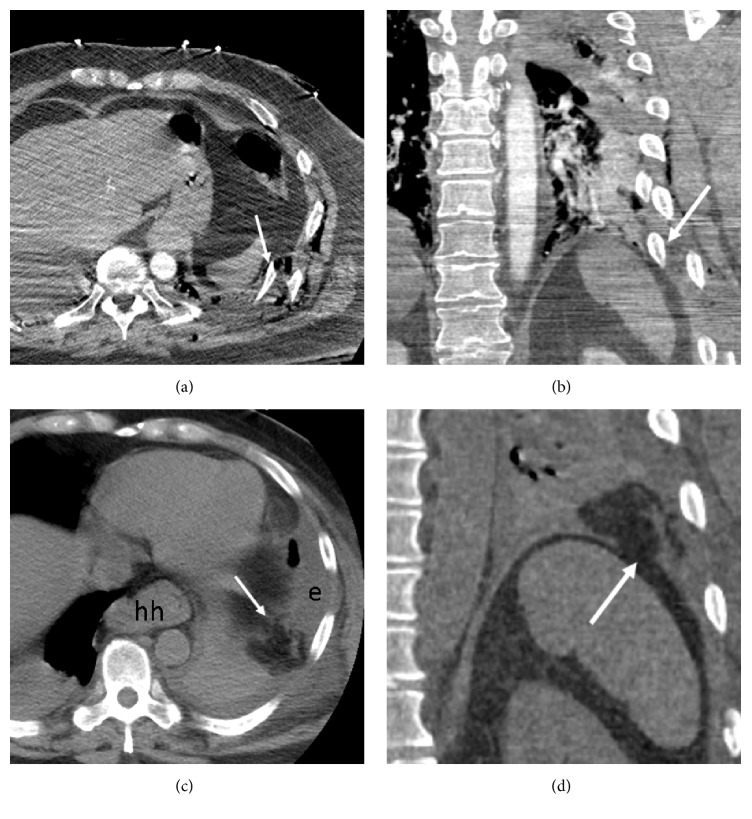
Fractured rib sign in a 64-year-old man after blunt trauma. (a, b) Contrast-enhanced trauma chest MDCT (axial and coronal planes) shows displaced left lower rib fractures abutting left diaphragm (arrows). The diaphragm appears intact. A small hiatal hernia is present (hh). (c, d) Repeat MDCT 4 days later (axial and coronal planes) shows new herniated omental fat (arrows) within left hemithorax due to subacute diaphragm rupture. Note discontinuity of the diaphragm (d). The large left effusion (e) is new.

A 64-year-old male restrained driver suffered blunt chest and abdominal trauma following a motor vehicle collision. He sustained a large left lung contusion, tiny left pneumothorax, fractures of the pelvis and lumbar transverse processes, and multiple comminuted ribs fractures. He did not require intubation. Contrast-enhanced trauma MDCT better depicted multiple displaced compound fractures of the left 3rd through the 11th ribs in close proximity to the diaphragm, but the diaphragm appeared intact (Figures 2(a) and 2(b)). In addition, left lung contusion and tiny left pneumothorax were present.

A repeat MDCT was performed four days later to evaluate an enlarging left pleural effusion. CT depicted new herniation of omental fat into the left chest, compatible with evolving TDR (Figures 2(c) and 2(d)). Serosanguinous pleura fluid was drained following left chest tube placement. Exploratory laparotomy on the same day revealed that the left 10th-rib fracture pierced the diaphragm with mildly edematous omental fat herniating through the 5 cm diaphragmatic laceration. The omentum was easily reduced, and the diaphragmatic laceration was surgically closed. His recovery was otherwise uneventful.

## 4. Discussion

Up to 8% of patients with severe blunt thoracoabdominal trauma develop a traumatic diaphragmatic injury [1]. TDR is rarely an isolated injury, and affected patients typically have a high injury severity score. While TDR represents only 5% of all diaphragmatic hernias, it is responsible for 90% of hernias that eventually become incarcerated, and most manifest within three years of the injury [1]. In a number of cases, TDR can present years later after the trauma and carry a mortality rate of 30–60% [7]. Motor vehicle collisions are responsible for up to 90% of TDR, with the remainder due to falls or crush or penetrating injuries [1, 8]. Left-sided diaphragmatic injuries are typically more clinically apparent and symptomatic [8]. The liver likely has a protective effect on the right diaphragm, and right diaphragmatic injuries may be underdiagnosed [1, 8].

It has been reported that the diagnosis of TDR may be missed initially in up to 30% of cases on MDCT [3]. Affected patients typically have severe multisystem injures that may overshadow the diagnosis of TDR, and there may be lack of awareness of the various imaging signs of diaphragmatic injury [1, 8]. In some cases, like ours, herniation of abdominal contents likely develops after the first trauma assessment. While a penetrating diaphragmatic injury from a rib fracture is typically small and may be initially inconspicuous, it will likely enlarge over time as negative intrathoracic pressure with inspiration gradually promotes herniation of abdominal contents into the chest [2, 8]. When a patient with TDR is intubated following blunt trauma, positive pressure ventilation may prevent the herniation of abdominal contents into the chest, and the diagnosis may only become apparent after extubation [1]. Subacute rupture is possible if diaphragmatic tissue is devitalized at the time of injury and subsequently breaks down. Because omentum is pliable and mobile, it may be the first abdominal structure to herniate into the chest [1, 7].

Spontaneous closure of TDR has never been reported, and almost all cases should be surgically repaired [1]. Early diagnosis is important because small laceration is typically easier to repair. A large TDR is inherently more complicated and may be associated with dense thoracic and abdominal adhesions.

In all cases with significant blunt thoracoabdominal trauma, thorough evaluation is required to exclude TDR, and follow-up evaluation should be considered to assess potential delayed development of TDR [9]. In difficult cases, MR imaging may secure the diagnosis of TDR but may not always be well suited for multitrauma patients [1, 8]. When the index of suspicion is high for diaphragmatic injury but imaging studies are inconclusive, laparoscopic or thoracoscopic exploration may confirm or exclude TDR.

Farboud et al. reported a 77-year-old man who sustained fractures of the left 3rd to 10th ribs and a left diaphragmatic hernia after falling 8 feet from a ladder. Urgent left thoracotomy revealed that the sharp edge of the displaced left 7th-rib fracture had pierced the diaphragm, and omentum had herniated into the chest through the diaphragmatic defect. Following resection of the rib fragment, the omentum was reduced, the diaphragmatic defect was repaired, and the patient recovered uneventfully [10]. Holland and Quint reported a case of left diaphragm laceration adjacent to rib fractures. CT performed 4 hours after the trauma revealed left hemopneumothorax, fractures of several left lower lateral ribs, and herniation of omentum into chest [5].

In our cases, both patients had the subacute diagnosis of a small TDR from a penetrating rib injury, with herniation of omental fat within two weeks following trauma. Both had the fractured rib sign on initial CT, with rib fracture fragments protruding into the chest cavity in close proximity to the diaphragm. In this setting, radiologists should have a high index of suspicion for TDR. Intrathoracic omental fat, the CT “funky fat” sign, should also alert the radiologist to search for TDR and may be a delayed finding. In our cases, early diagnosis of TDR likely prevented strangulation of omentum and subsequent development of a large hernia sac containing abdominal visceral organ.
